# 粒细胞样髓源性抑制细胞在非小细胞肺癌中的研究进展

**DOI:** 10.3779/j.issn.1009-3419.2023.106.28

**Published:** 2024-01-20

**Authors:** Chaodan YANG, Rui ZHU, Yuting ZHANG, Lisha YING, Jiamin WANG, Pan LIU, Dan SU

**Affiliations:** ^1^310024 杭州，国科大杭州高等研究院分子医学院（杨朝丹，王佳敏，苏丹）; ^1^School of Molecular Medicine, Hangzhou Institute for Advanced Study, University of Chinese Academy of Sciences, Hangzhou 310024, China; ^2^201203 上海，中国科学院上海药物研究所（杨朝丹，王佳敏）; ^2^Shanghai Institute of Materia Medica, Chinese Academy of Sciences, Shanghai 201203, China; ^3^100049 北京，中国科学院大学（杨朝丹，王佳敏）; ^3^University ofChinese Academy of Sciences, Beijing 100049, China; ^4^310022 杭州，浙江省肿瘤医院病理科，中国科学院杭州医学研究所（朱蕊，应莉莎，刘盼，苏丹）; ^4^Department of Pathology, Zhejiang Cancer Hospital, Hangzhou Instituteof Medicine, Chinese Academy of Sciences, Hangzhou 310022, China; ^5^310022 杭州，温州医科大学研究生培养基地，浙江省肿瘤医院（张煜婷）; ^5^Postgraduate Training Base Alliance of Wenzhou MedicalUniversity, Zhejiang Cancer Hospital, Hangzhou 310022, China

**Keywords:** 肺肿瘤, 粒细胞样髓源性抑制细胞, 免疫微环境, 免疫治疗, 靶向治疗, Lung neoplasms, Granulocytic myeloid-derived suppressor cells, Immune microenvironment, Immunotherapy, Targeted therapy

## Abstract

粒细胞样髓源性抑制细胞（granulocytic myeloid-derived suppressor cells, G-MDSCs）是MDSCs的主要亚群之一，在多数癌症中广泛富集，通过表达精氨酸酶1（arginase-1, Arg-1）及活性氧（reactive oxygen species, ROS）等机制抑制淋巴T细胞杀伤功能，重塑肿瘤免疫微环境，促进肿瘤的发生发展。近年来，越来越多的研究发现G-MDSCs与非小细胞肺癌患者的预后和免疫治疗疗效具有显著相关性，使用特异性靶向G-MDSCs的募集、分化和功能的药物能够有效抑制肿瘤的进展。本文主要就G-MDSCs在非小细胞肺癌中的免疫抑制作用及其相关通路靶向药物的研究进展进行综述。

## Abstract (en)

Granulocytic myeloid-derived suppressor cells (G-MDSCs) are one of the main subgroups of MDSCs, which are widely enriched in most cancers. It can inhibit the killing function of T-lymphocyte through the expression of arginase-1 (Arg-1) and reactive oxygen species (ROS), reshape the tumor immune microenvironment, and promote the occurrence and development of tumors. In recent years, more and more studies have found that G-MDSCs are significantly correlated with the prognosis and immunotherapy efficacy of patients with non-small cell lung cancer, and the use of drugs specifically targeting the recruitment, differentiation and function of G-MDSCs can effectively inhibit tumor progression. This article reviews the immunosuppressive effect of G-MDSCs in non-small cell lung cancer and the progress of related pathway targeting drugs.

肺癌是一种起源于肺部气管、支气管黏膜或腺体的恶性肿瘤。美国临床肿瘤杂志发布的2020全球癌症统计^[1]^显示，肺癌是男性第一高发的癌症类型，占总病例的14.3%，也是男性癌症相关死亡的第一大原因；在女性癌症患者中肺癌发病率排名第三，死亡人数占女性癌症死亡总数的13.7%，仅次于乳腺癌，排名第二。肺癌患者中非小细胞肺癌（non-small cell lung cancer, NSCLC）患病人数占整体肺癌患者的85%左右^[2]^。随着分子靶向治疗及免疫治疗的兴起，许多分子靶向药物和免疫治疗药物已经成功应用于NSCLC的临床治疗，例如表皮生长因子受体抑制剂、免疫检查点抑制剂等^[3]^。然而，多数分子靶向药物在治疗过程中易产生耐药性，患者可能出现疗效不佳、治疗周期延长和毒副作用增大等问题^[4]^。因此，寻找NSCLC的新型分子治疗靶点并开发相应的抑制剂是当前亟需解决的重要问题。

近年来，研究人员对肿瘤微环境（tumor micro-environment, TME）在肿瘤发生发展中的调控机制越来越重视。髓源性抑制细胞（myeloid-derived suppressor cells, MDSCs）在TME中发挥免疫抑制功能，其中粒细胞样MDSCs（granulocytic MDSCs, G-MDSCs）是MDSCs中最主要的细胞亚群，对NSCLC和其他多种恶性肿瘤的发生发展有重要影响^[5]^。因此，针对G-MDSCs相关通路的靶向治疗可能为NSCLC的精准治疗提供新机遇。

## 1 G-MDSCs的生成

根据细胞表型和基因表达谱的差异，MDSCs主要分为粒细胞来源的G-MDSCs和单核细胞来源的单核细胞样MDSCs（monocytic MDSCs, M-MDSCs）。G-MDSCs也被称作多核MDSCs（polymorphonuclear MDSCs, PMN-MDSCs），它是中性粒细胞分化异常而产生的一种具有免疫抑制功能的G-MDSCs，人类G-MDSCs在细胞表型上被定义为CD14^-^CD11b^+^CD33^+^CD15^+^/CD66b^+^，小鼠中G-MDSCs表型被定义为CD11b^+^Ly6C^lo^Ly6G^+^^[6]^。M-MDSCs是单核巨噬细胞生成过程出现异常分化而形成的M-MDSCs，人类细胞表型表现为CD14^+^HLA^-^DR^-/lo^，在小鼠中被定义为CD11b^+^Ly6C^hi^Ly6G^-^^[7]^。在大多数类型的肿瘤中，G-MDSCs占MDSCs总数的70%-80%，而M-MDSCs通常不超过20%，这表明骨髓细胞调控这两个亚群生成的分子机制存在差异^[8]^。

G-MDSCs与具有细胞免疫功能的中性粒细胞有着共同的起源。在正常生理条件下，骨髓中的造血干细胞分化为髓细胞，最终分化为单核细胞和粒细胞，其中中性粒细胞属于粒细胞亚群^[9]^。成熟的中性粒细胞寿命较短，在病原体入侵机体时，通过粒细胞集落刺激因子（granulocyte colony-stimulating factor, G-CSF）、CXC-趋化因子受体（CXC-chemokine receptor, CXCR）和CXC-趋化因子配体（CXC-chemokine ligand, CXCL）等信号的作用，从骨髓不断生成并迁移至外周血，发挥免疫效应。然而，在癌症中，骨髓细胞受到微弱而持久的刺激信号，长期暴露于肿瘤特异的微环境中，导致其分化所需的转录因子功能紊乱，从而破坏了正常的分化途径。在中性粒细胞向成熟分化的过程中，由于分化受阻，形成的未成熟中性粒细胞为G-MDSCs。此类G-MDSCs不再具备中性粒细胞的细胞免疫功能，相反，它们表现出免疫抑制效应^[10]^。在肿瘤形成的过程中，中性粒细胞也会转化为G-MDSCs，参与G-MDSCs的积累^[11]^。

## 2 G-MDSCs促进NSCLC恶性进展的分子机制

G-MDSCs在NSCLC患者的预后预测和免疫治疗监测方面具有重要的作用。G-MDSCs作为一种免疫抑制性细胞类型，可以抑制免疫系统对肿瘤的攻击，从而促进肿瘤的生长和转移。研究^[12⇓-14]^表明，原发性或转移性肺癌患者外周血中存在大量的G-MDSCs，G-MDSCs的富集程度与NSCLC患者的总生存期、肿瘤原发灶-淋巴结-转移（tumor-node-metastasis, TNM）分期及淋巴结转移密切相关。外周血和肺肿瘤组织中高水平的G-MDSCs往往伴有自然杀伤（natural killer, NK）细胞比例的减少，二者存在情况呈负相关^[15]^。因此，高水平的G-MDSCs提示患者的不良预后。

此外，G-MDSCs的水平还与免疫治疗的疗效密切相关，在对接受了靶向程序性死亡配体1（programmed death ligand 1, PD-L1）免疫治疗的NSCLC患者的外周血检测中发现，其G-MDSCs会表现出更高的PD-L1表达水平^[16]^。高水平的G-MDSCs可以抑制免疫治疗的效果，降低患者的生存率。因此，通过监测G-MDSCs的水平，可以评估患者的预后及其对免疫治疗的响应，并为治疗策略的选择提供指导。

### 2.1 影响T细胞功能

T细胞是免疫系统杀伤肿瘤的重要武器，当其增殖及活化受到抑制时，免疫系统抗击肿瘤的能力会大幅下降。研究^[17]^发现，MDSCs会影响T细胞的功能，从而发挥促肿瘤效应。MDSCs的2个主要细胞亚群对抑制T细胞功能的调控机制存在差异。G-MDSCs表达高水平的活性氧（reactive oxygen species, ROS）和低水平的一氧化氮（nitric oxide, NO），而M-MDSCs则相反。2个亚群都表达精氨酸酶1（arginase-1, Arg-1）^[18]^。G-MDSCs主要通过以下2种代谢方式影响T细胞功能。

#### 2.1.1 产生Arg-1

G-MDSCs会产生Arg-1消耗对控制T细胞功能至关重要的L-精氨酸（L-arginine, L-Arg）^[19]^。T细胞表面受体CD3分子负责将T细胞表面受体（T cell receptor, TCR）识别抗原时产生的活化信号转导至T细胞内部^[20]^，CD3ζ链是CD3分子的组成肽链之一。NSCLC患者中G-MDSCs产生过量Arg-1会消耗L-Arg，导致T细胞表面的CD3ζ链表达下降，进而干扰T细胞信号传导，影响后续的细胞免疫功能^[21]^。L-Arg缺乏时会促进G-MDSCs的积累，进一步抑制T细胞抗肿瘤反应^[22]^。聚乙二醇化形式的分解代谢酶精氨酸酶I（pegylated form of the catabolic enzyme arginase I, peg-Arg I）升高会导致G-MDSCs积累，peg-Arg I还参与阻断正常活化T细胞的糖酵解途径，致使T细胞功能受到抑制^[22]^。

#### 2.1.2 产生ROS

NSCLC患者外周血G-MDSCs特异性高表达ROS^[21]^。大量ROS的积累会导致氧化应激，阻碍细胞蛋白质合成及翻译过程^[23]^；高水平的ROS同样可以降低TCR链的表达，抑制T细胞的活化^[24]^；ROS的增加还可以消除CD8^+ ^T细胞的抗原特异性反应^[21,25]^。此外，ROS还参与缺氧诱导因子1α（hypoxia-inducible factor-1α, HIF-1α）的活化，HIF-1α调节M-MDSCs向M2型巨噬细胞分化^[26,27]^，M2型巨噬细胞抑制1型辅助T细胞（type 1 helper T cell, Th1）的免疫应答，促进Th2的免疫应答^[28]^，这打破了机体中Th1和Th2相对平衡的状态，因此大多数情况下，M2表型癌症患者的总生存率较差。

### 2.2 影响NK细胞功能

NK细胞作为固有免疫应答系统中的重要成员，在病原体入侵机体的早期就发挥重要的免疫杀伤作用。临床研究^[15]^数据显示，原发性或转移性肺肿瘤患者TME及外周血中G-MDSCs介导的ROS和Arg-1的生成会损伤NK细胞的功能，导致NK细胞数量减少，进而抑制机体固有免疫应答。G-MDSCs来源的外泌体内含有的一种微小RNA（microRNAs, miRNAs），会造成NK细胞溶解，破坏NK细胞的完整性^[28]^。NSCLC患者中G-MDSCs高水平表达5’-核苷酸酶（又称CD73），其产生的腺苷是一种免疫抑制分子，通过限制肿瘤坏死因子α（tumor necrosis factor α, TNF-α）等细胞因子的释放抑制NK细胞的细胞毒性^[29]^。

除了影响免疫细胞功能外，NSCLC会分泌高水平的趋化因子招募G-MDSCs进入TME^[30]^。在TME中，G-MDSCs反过来也会分泌趋化因子、转化生长因子β、白细胞介素-10等多种细胞因子诱导肿瘤细胞上皮-间充质转化，促进肿瘤的转移^[31]^。

## 3 NSCLC中靶向G-MDSCs的治疗方式

G-MDSCs在NSCLC患者的预后指示和免疫治疗监测方面显示出很强的相关性，但其在临床应用中仍需要进一步的研究和验证，针对G-MDSCs募集、分化和功能的治疗方式的开发是十分有必要的。

### 3.1 抑制G-MDSCs的扩增和募集

#### 3.1.1 靶向转录激活因子3（signal transducer and activator of transcription 3, STAT3）通路

在NSCLC中，驱动G-MDSCs扩增的主要信号分子之一是STAT3^[32]^。激活STAT3可通过促进抗凋亡基因的表达，抑制G-MDSCs凋亡，阻止G-MDSCs向成熟细胞分化；NADPH氧化酶2（NADPH oxidase, NOX2）的活性上调介导G-MDSCs中ROS产生增多，NOX2活性缺失的G-MDSCs会失去抑制T细胞反应的能力，G-MDSCs中NOX2的表达受STAT3控制^[33]^；STAT3信号通路还通过促进Arg-1表达来增强G-MDSCs的功能^[34]^。因此，靶向STAT3信号通路是一种有效的破坏G-MDSCs的发育和功能的治疗策略^[35]^，进而克服G-MDSCs积累和扩增导致的免疫抑制。中药泽漆汤是临床上常用于治疗NSCLC的一种中药，其机制之一就是靶向STAT3通路诱导G-DMSCs大量凋亡，阻止G-DMSCs在TME中的大量扩增和聚集^[36]^。

#### 3.1.2 抑制G-MDSCs的代谢

在G-MDSCs存在的TME中，NSCLC细胞会通过代谢重编程将营养物质产生的能量最大限度地用于肿瘤发展，此过程也将加剧G-MDSCs的积累与扩增，进一步抑制T细胞的免疫功能^[37,38]^。因此，靶向G-MDSCs的代谢途径也是抑制G-MDSCs扩增的手段之一。

表达G-MDSCs的NSCLC患者和Lewis小鼠肺癌模型均出现溶酶体酸性脂肪酶（lysosomal acid lipase, LAL）降低的现象，LAL缺失（deficiency of LAL, LAL-D）也会诱导髓系细胞分化为G-MDSCs，并促进肿瘤生长和转移。阻断糖酵解中的丙酮酸脱氢酶（pyruvate dehydrogenase, PDH）可以逆转LAL-D的G-MDSCs的免疫抑制能力，也会减少ROS的过度产生。另外，LAL可以单独或与抗PD-L1免疫疗法联合用于治疗NSCLC患者^[37]^。

腺苷是细胞代谢中三磷酸腺苷衍生的核苷，在Lewis小鼠肺癌模型中A_2B_腺苷受体在造血细胞上的表达会促进G-MDSCs优先扩增，G-MDSCs在TME中产生的腺苷有助于其发挥免疫抑制效应，因此，腺苷抑制剂也可以抑制G-MDSCs的扩增^[39,40]^。

#### 3.1.3 抑制募集G-MDSCs所需的趋化因子

癌症相关成纤维细胞（cancer-associated fibroblasts, CAF）会分泌粒细胞特异性的CXCL1作用于G-MDSCs上的CXCR2，导致G-MDSCs被募集到肿瘤部位，而CSF1可以抑制CXCL1的表达。在NSCLC患者外周血的检测中，CSF1高分泌的患者外周血中检测到的G-MDSCs数量很少。靶向CSF1受体的抑制剂与CXCR2拮抗剂联合使用可阻断小鼠肺癌模型中G-MDSCs向TME浸润^[41]^。探究CAF、G-MDSCs和免疫细胞相互作用在NSCLC对抗程序性死亡受体-1（programmed cell death 1, PD-1）/PD-L1治疗耐药中的作用也已进入临床研究阶段（NCT06024538）。

CXCR2在G-MDSCs向TME浸润的信号传递过程中也扮演着重要角色^[42]^，在Lewis小鼠肺癌模型中抑制CXCR2可达到抑制G-MDSCs募集的目的^[43]^。目前，CXCR2抑制剂SX682正在多项临床试验中（NCT04477343、NCT04574583和NCT03161431）。

### 3.2 诱导G-MDSCs分化

全反式维甲酸（all-trans retinoic acid, ATRA）是维生素A的衍生物，最早用于治疗急性早幼粒细胞白血病^[44]^。研究^[45]^发现ATRA可诱导癌症患者和荷瘤小鼠中G-MDSCs的分化，导致G-MDSCs失去活性从而改善机体的免疫反应。使用ATRA靶向G-MDSCs分化这一策略已在转移性黑色素瘤中展开I/II期临床试验，治疗期间患者外周血中循环的G-MDSCs数量大幅降低并且分化成为成熟的髓系细胞^[46]^。ATRA促使G-MDSCs分化的机制之一是增加谷胱甘肽生成所需的谷胱甘肽合酶，使谷胱甘肽积累，谷胱甘肽增多会诱导G-MDSCs分化为巨噬细胞和树突状细胞^[47]^；ATRA也会中和ROS^[48]^，ROS减少已被证实可促进G-MDSCs向巨噬细胞分化^[49]^。

LKB1缺陷NSCLC的转基因小鼠模型证明了肿瘤中的LKB1丢失会导致TME中G-MDSCs的富集，G-MDSCs在LKB1缺陷的NSCLC中的积累可以通过ATRA治疗来逆转，使肿瘤对免疫治疗敏感^[50]^。

### 3.3 抑制G-MDSCs的功能

消除Arg-1是抑制G-MDSCs功能的首要靶点。在Lewis小鼠肺癌模型中，前列腺素E2（prostaglandin E2, PGE2）已被证明可以诱导G-MDSCs上调Arg-1表达。环氧合酶2（cyclooxygenase-2, COX2）则是产生PGE2所必需的酶，因此，使用COX2抑制剂可以下调G-MDSCs中Arg-1的表达，抑制G-MDSCs的功能，从而达到抗肿瘤的目的^[51,52]^。非甾体抗炎药是一类主要抑制COX活性的药物，COX活性也会降低PG的产生^[53]^，根据一项检索了自1980年以来进行的流行病学研究^[54]^的分析，发现定期使用非甾体抗炎药可以有效降低肺癌的发病率。

消除ROS是抑制G-MDSCs功能的另一条重要途径，ROS抑制剂可有效减少小鼠中G-MDSCs介导的免疫抑制^[55]^，许多靶向G-MDSCs的抑制剂都直接或间接地抑制ROS。在Lewis荷瘤小鼠中脂肪酸转运蛋白2（fatty acid transporter 2, FATP2）通过控制ROS的产生调节MDSCs的免疫抑制功能，抑制剂Lipofermata可以通过阻断MDSCs中FATP2的表达减少脂质积累和ROS，降低MDSCs的免疫抑制活性，抑制小鼠肿瘤生长^[56]^。

TME中的G-MDSCs处于缺氧环境，此时升高的HIF-1α上调G-MDSCs中浆细胞瘤变异易位基因1（plasmacytoma variant translocation 1, PVT1）的表达，PVT1表达降低后G-MDSC释放的Arg-1与ROS均下降，T细胞抗肿瘤能力得以恢复。使用HIF-1α的抑制剂YC-1作用于Lewis肺癌细胞后，G-MDSCs中PVT1的表达明显降低，表明靶向PVT1可以减弱G-MDSC介导的免疫抑制，这可能是一种潜在的治疗策略^[57]^。

### 3.4 促进G-MDSCs清除

内质网（endoplasmic reticulum, ER）应激是细胞应对缺氧、营养匮乏等不利于细胞生存的情况下所产生的一种保护性应激反应，该通路被激活后会诱导应激细胞死亡，恢复和维持ER稳态^[58]^。G-MDSCs的显著特征之一是ER应激相关基因上调，癌症患者的G-MDSCs会表现出更高水平的ER应激。ER应激反应被激活还会上调LOX-1的表达，将正常的中性粒细胞转化为G-MDSCs^[11]^。癌症患者体内G-MDSCs的寿命受到TNF相关的凋亡诱导配体及其受体（TNF-related apoptosis-inducing ligand-receptor, TRAIL-R）的调控。TRAIL-R组成成分中的诱饵受体1（decoy receptor 1, DCR1）和DCR2在G-MDSCs表面的表达远低于中性粒细胞，这两种DER可拮抗TRAIL介导的凋亡信号，二者表达降低导致G-MDSCs对TRAIL信号敏感。ER应激诱导剂Tunicamycin会使人类G-MDSCs表面的DCR1和DCR2明显下调，类似于癌症患者中观察到的G-MDSCs的情况^[59]^。G-MDSCs所产生的ROS也是一种ER应激反应的诱导剂^[60,61]^。这提示使用ER应激抑制剂靶向G-MDSCs表面的TRAIL-R可提供一条清除G-MDSCs的途径^[62]^。

常规化疗药物如紫杉醇、5-氟尿嘧啶及吉西他滨等都已被证实能有效减少外周血中G-MDSCs的数量^[5]^。吉西他滨已进入NSCLC IIIB期临床研究（NCT03302247）。免疫检查点抑制剂与G-MDSCs抑制剂联合用药治疗原发和转移性肿瘤也收获了显著的治疗效果^[41,63,64]^。

## 4 小结与展望

G-MDSCs在NSCLC TME中的免疫抑制效应及其在肿瘤发生发展中的独特作用提示其作为肿瘤靶向治疗一类潜在药物靶点的可能性。但是这一细胞亚群仍存在一定争议性和局限性：首先，G-MDSCs与发挥抑制作用的中性粒细胞没有一个标准的定义^[65]^；其次，G-MDSCs因为在MDSCs中数量占比巨大而一直备受关注，但近年来的研究^[66,67]^发现另一细胞亚群M-MDSCs在肿瘤进展中同样有着重要的意义，在NSCLC的研究中也有文献^[68]^报道外周血中M-MDSCs水平高的NSCLC患者预后更差，在一项关于22例NSCLC患者的探索性分析^[69]^中发现高水平M-MDSCs与免疫治疗的原发性耐药性密切相关，这提示NSCLC中同时靶向这两个细胞亚群的治疗或许获益更高；再者，NSCLC中靶向G-MDSCs的部分治疗方式同样也靶向M-MDSCs，例如有文献^[70]^报道肿瘤相关成纤维细胞（cancer-associated fibroblasts, CAFs）在NSCLC中通过诱导M-MDSCs来促进免疫抑制。

MDSCs在TME中发挥免疫抑制作用的重要性已经毫无争议，G-MDSCs和M-MDSCs在细胞表型、基因表达和 调控通路上存在明显差异，这提示研究者和临床医生应注意二者的差异性以达到精准医疗的治疗效果。但若在NSCLC患者中出现MDSCs整体表达升高时，治疗手段上单一靶向某一免疫抑制细胞亚群可能不足以控制肿瘤进展。总的来说，联合治疗策略是延缓或逆转患者耐药最重要且最有效的措施，目前在包括NSCLC在内的多种癌症中，传统化疗药物、免疫治疗药物与G-MDSCs抑制剂的联用，可能成为分子靶向治疗及免疫治疗时代背景下，改善患者预后的有效治疗方案之一。
